# A Novel Brain MRI Image Segmentation Method Using an Improved Multi-View Fuzzy *c*-Means Clustering Algorithm

**DOI:** 10.3389/fnins.2021.662674

**Published:** 2021-03-25

**Authors:** Lei Hua, Yi Gu, Xiaoqing Gu, Jing Xue, Tongguang Ni

**Affiliations:** ^1^School of Artificial Intelligence and Computer Science, Jiangnan University, Wuxi, China; ^2^School of Computer and Artificial Intelligence, Changzhou University, Changzhou, China; ^3^Department of Nephrology, The Affiliated Wuxi People’s Hospital of Nanjing Medical University, Wuxi, China

**Keywords:** brain magnetic resonance imaging, multi-view learning, fuzzy clustering, adaptive learning, image segmentation

## Abstract

**Background:** The brain magnetic resonance imaging (MRI) image segmentation method mainly refers to the division of brain tissue, which can be divided into tissue parts such as white matter (WM), gray matter (GM), and cerebrospinal fluid (CSF). The segmentation results can provide a basis for medical image registration, 3D reconstruction, and visualization. Generally, MRI images have defects such as partial volume effects, uneven grayscale, and noise. Therefore, in practical applications, the segmentation of brain MRI images has difficulty obtaining high accuracy.

**Materials and Methods:** The fuzzy clustering algorithm establishes the expression of the uncertainty of the sample category and can describe the ambiguity brought by the partial volume effect to the brain MRI image, so it is very suitable for brain MRI image segmentation (B-MRI-IS). The classic fuzzy *c*-means (FCM) algorithm is extremely sensitive to noise and offset fields. If the algorithm is used directly to segment the brain MRI image, the ideal segmentation result cannot be obtained. Accordingly, considering the defects of MRI medical images, this study uses an improved multiview FCM clustering algorithm (IMV-FCM) to improve the algorithm’s segmentation accuracy of brain images. IMV-FCM uses a view weight adaptive learning mechanism so that each view obtains the optimal weight according to its cluster contribution. The final division result is obtained through the view ensemble method. Under the view weight adaptive learning mechanism, the coordination between various views is more flexible, and each view can be adaptively learned to achieve better clustering effects.

**Results:** The segmentation results of a large number of brain MRI images show that IMV-FCM has better segmentation performance and can accurately segment brain tissue. Compared with several related clustering algorithms, the IMV-FCM algorithm has better adaptability and better clustering performance.

## Introduction

The brain is the human body’s nerve center, controlling people’s thinking, memory, speech, and movement, and plays a role in regulating human organs. When the brain is healthy, it can work quickly and efficiently. However, when something goes wrong, the result can be devastating. In recent years, the problem of brain diseases has become increasingly prominent due to comprehensive factors such as high pressure in people’s lives, fast-paced activities, extreme tension in thoughts and emotions, frequent accidents, and serious aging in the population, which continue to threaten people’s health. In clinical medicine, doctors usually use MRI technology to diagnose brain diseases. MRI is a very advanced medical imaging technology. It visualizes the structure and function of the human body through radiology and is particularly suitable for brain tissue research. By using this technology, high soft tissue contrast can be obtained, and it has the advantages of noninvasiveness, nonradiation, and high-precision spatial resolution (Ji et al., 2011; Jiang et al., 2012; Thanh and Wu, 2013).

Neuroscience researchers need to segment brain MRI images to quantitatively study brain diseases. B-MRI-IS marks each pixel in the image as the corresponding brain tissue anatomical structure, such as the thalamus, hippocampus, and ventricles. Then, the segmented tissue size, shape, location, and other characteristics are used to evaluate and formulate medical plans. Experiments have shown that the abnormal shape or volume of certain anatomical regions of the brain is related to brain diseases, such as Alzheimer’s disease and Parkinson’s disease. The quality of B-MRI-IS determines the reliability of researchers’ assessment of brain diseases. The segmentation quality is determined by the segmentation method, so the segmentation method is very important in the entire diagnosis process. Usually, accurate segmentation results of brain tissue are obtained by manual segmentation by experienced brain experts. However, when faced with a large number of datasets, manual segmentation methods become quite expensive, time consuming, and impractical. Moreover, due to differences in experience and knowledge among experts, the segmentation results are not uniform. Therefore, the use of more accurate B-MRI-IS algorithms to quantitatively analyze the volume shape of each tissue in brain MRI images has become the focus of medical image research.

Traditional image segmentation methods include threshold-based methods, clustering-based methods, region-based methods, edge-based methods, and graph theory-based methods (Gu et al., 2019; Kumar et al., 2020). Considering the problems in the segmentation process, scholars have proposed their solutions. Among them, fuzzy clustering algorithms are widely used. The literature (Bezdek, 1981) proposed the FCM algorithm for the first time, introducing the concept of ambiguity into the clustering method (Li et al., 2014), and proposed an optimized bias field estimation and tissue segmentation method. Through the simultaneous iteration of bias field estimation and tissue segmentation, the final segmentation accuracy is improved, and the algorithm is very robust to initialization. The literature (Chuang et al., 2006) proposed the FCM algorithm based on spatial relationships. The algorithm integrates the spatial information of the sum of membership degrees of all points in the window into the traditional FCM. Experiments show that the improved algorithm has certain robustness to noise. Noise interference has always been one of the important interference factors in medical image segmentation. Due to the complexity of the human brain tissue structure and the imaging mechanism of MRI technology, MRI images will show phenomena such as strong noise, false images, and weak boundaries. For example, traditional active contour models often only use target edge information, which leads to premature stopping when processing strongly noisy images, and weak boundary leakage occurs when the boundary is blurred. Many studies use grayscale information and spatial information to suppress noise interference. The literature (Ahmed et al., 2002) introduces local spatial terms into the objective function of FCM to make the algorithm perform better. The literature (Chen and Zhang, 2004) replaces the traditional Euclidean distance with the core distance to make the algorithm more robust. The literature (Krinidis and Chatzis, 2010) proposed a new blur factor and introduced the kernel distance, which is more robust to noise. The literature (Gong et al., 2013) proposed a kernel-based adaptive regularization FCM algorithm. This method introduces adaptive parameters and uses mean filtering, median filtering, and custom filtering of images. There is no need to calculate parameters for each iteration, which greatly saves time and cost. Additionally, the robustness of the algorithm to noise is improved. The literature (Elazab et al., 2015) improves the quality and compression rate of the reconstructed image by compressing the image in blocks.

**FIGURE 1:**
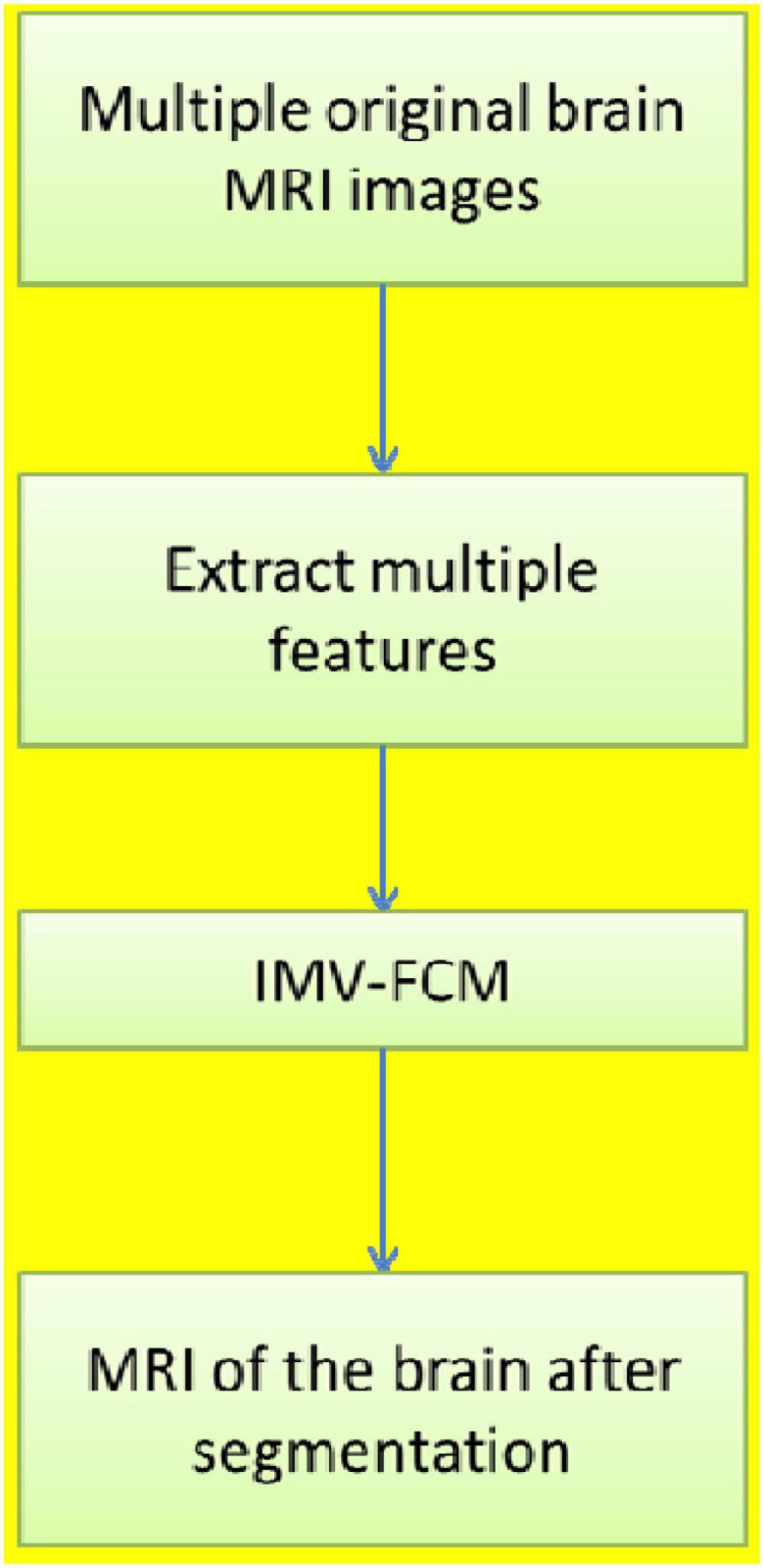
The motivation for this research.

Figure 1 shows the motivation for this research. To reduce the influence of noise in the segmentation process, the accuracy of B-MRI-IS is improved. This article uses an improved multiview FCM algorithm. The work in this paper is summarized as follows:

1. The B-MRI-IS process is proposed. First, the original brain MRI image is preprocessed, such as denoising. Second, the histogram of oriented gradient (HOG) feature, entropy feature, gradient feature, and contrast feature of the original image are extracted. Each feature data point is used as view data to construct multiview data (MVD). Third, the constructed MVD is input into the clustering model to obtain the final clustering result. The clustering result is the image segmentation result.
2. Based on multiview FCM, the IMV-FCM algorithm is proposed. IMV-FCM uses a view weight adaptive learning mechanism so that each view obtains the optimal weight according to its cluster contribution. The final division result is obtained through the view ensemble method. Under the view weight adaptive learning mechanism, the coordination between various views is more flexible, and each view can be adaptively learned to achieve better clustering effects.
3. To demonstrate the effectiveness of the clustering model used in this paper, single-view clustering and multiview clustering algorithms are used for experimental comparison. To demonstrate the antinoise of the algorithm, after adding noise to the experimental image, each algorithm is used for clustering comparison. The results show that the algorithm used in this paper is the most robust to noise compared with other comparison algorithms and can accurately segment brain tissue.

## Backgrounds

### MRI Technology

In medicine, human brain tissue includes the cerebrum, cerebellum, and brainstem, each of which has relatively independent characteristics. At the same time, the skull structure can be divided into white matter (WM), gray matter (GM), and cerebrospinal fluid (CSF) (Wang et al., 2015). GM contains WM, and CSF fills the crooked groove formed by the GM folds. The brain tissue images show that the gray levels of various brain tissues change slowly and are not constant. Coupled with the influence of various noises, it is easy to cause the gray values to cross and overlap the internal brain tissues. The only correspondence is that one gray value may correspond to several brain internal tissues. In addition, internal brain images with different imaging morphologies also provide different information, and they appear as different gray levels on the images. According to the image gray level, it can be divided into four types, namely, white, off-white, gray, and black. Table 1 shows the characteristics of different gray levels.

**TABLE 1:** Image grayscale characteristics of adult brain tissue.

Organization	T1-weighted image	Pd weighted image	T2 weighted image
WM	Off-white	Gray	Black gray
GM	Gray	Off-white	Off-white
CSF	Black	Gray	White

The proton density ρ, longitudinal relaxation time T1, and transverse relaxation time T2 of brain tissue constitute the high-dimensional biophysical property space of the brain. The T1 and T2 parameters of normal and pathological tissues in different organs of the human body are fixed, and there are certain differences between them. Additionally, different tissues have different densities, and the differences in these properties between brain tissues are the biological basis of the solvability of brain tissue segmentation. Table 2 shows the relevant attributes of the main brain tissues in MRI images.

**TABLE 2:** Related attributes of main brain tissues in MRI images.

Tissue	T1 (s)	T2 (ms)	ρ (1.5)
CSF	0.8–20	110–2,000	70–230
WM	0.76–1.08	61–100	70–90
GM	1.09–2.15	61–109	85–125
Meninges	0.5–2.2	50–165	5–44
Muscle	0.95–1.82	20–67	45–90
Adipose	0.2–0.75	53–94	50–100

### Clustering Algorithm

**FIGURE 2:**
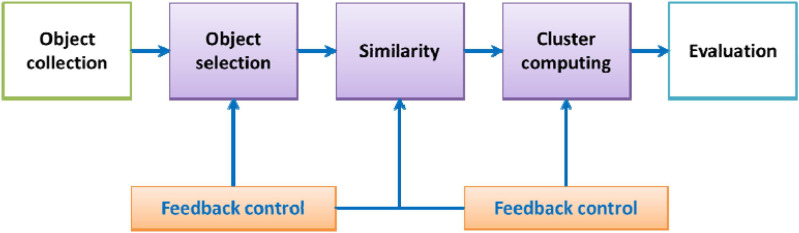
Cluster analysis flowchart.

These attributes are determined by factors such as the composition of the brain tissue and the local microstructure. Cluster analysis aims to make reasonable classifications according to the characteristics of the sample and divide the data points with similar characteristics into one category. The idea of the cluster analysis algorithm comes from taxonomy. As MRI images contain increasing information, the requirements for classification are increasing. People have introduced multivariate analysis into taxonomy, forming cluster analysis. The goal of cluster analysis is to collect data to classify when the data points are similar (Bezdek, 1980; Hall et al., 1992; Jiang et al., 2014; Qian et al., 2015, 2016, 2018; Zheng et al., 2015; Jiang et al., 2019; Jiang et al., 2020). Cluster analysis is a technique to study the relationship between sample data points. The clustering results reveal the internal connections and differences between sample data and provide an important basis for data processing. The cluster analysis process is shown in Figure 2.

The clustering object refers to the grayscale collection of image pixels. The similarity measure is a key step used to measure the similarity between data points. Euclidean distance is often used as the similarity measure. The clustering algorithm uses similarity measures to select data points that are very close to each other and classify them into a cluster. Additionally, the result of clustering will affect the selection of subsequent objects and the similarity measurement, so there is a feedback process. The last step of clustering is to evaluate the clustering results. The importance of evaluating the clustering results will not be lower than the importance of the previous steps. For example, in a cluster analysis method based on a threshold value, it is necessary to judge the classification result to guide the selection of the threshold value. In the cluster analysis method with the function as the criterion, the judgment or evaluation of the classification result can be used to guide the selection of the appropriate criterion function.

Clustering algorithms can be divided into partition-based (Jing et al., 2007; Zhu et al., 2009; Yu et al., 2012; Qian et al., 2017), hierarchy-based (Hall and Goldgof, 2011; Devi and Setty, 2018), density-based (Sunjana and Azizah, 2020; Yin et al., 2020), grid-based (Wang et al., 2019; Zhang et al., 2020), and model-based (Lee et al., 2018; Chrobak et al., 2020) algorithms. Due to the complexity of brain medical images, the boundaries and boundaries of different tissues in the brain are uncertain and unclear. This requires a method for reducing the unclearness. Fuzzy mathematics based on fuzzy sets can solve these uncertain and imprecise problems. The fuzzy clustering algorithm in the clustering algorithm is most suitable for B-MRI-IS. The typical fuzzy clustering algorithm is the FCM algorithm. The principle of image segmentation based on FCM is to use the uncertainty of the classification of image pixels. The degree of membership is used to describe this uncertainty, the distance relationship between pixels is described according to the objective function, and the best cluster center is selected. To describe the clustering algorithm more clearly, Table 3 shows the symbols used in the FCM algorithm.

**TABLE 3:** Description of related symbols in FCM.

Symbols	Description	Symbols	Description
*X*	Dataset	*x*_*j*_	The *j*th pixel
*C*	Number of clusters	*N*	Total number of image pixels
*u*_*ij*_	The membership degree of pixel *x*_*i*_ belonging to the *j*th cluster	*z*_*i*_	The *i*th cluster center
*d*_*ij*_	Euclidean distance from sample point *x*_*j*_ to cluster center*z*_*i*_	*m*	Fuzzy factor

Assume the image pixel dataset is *X* = {*x*_1_,*x*_2_,…,*x*_*N*_}, where *x*_*i*_ represents the gray value of the image pixel. FCM’s idea is to transform the image segmentation process into the optimization process of the feature function when the objective function converges to realize the fuzzy classification of pixel data. That is, the image segmentation problem is transformed into a clustering problem of dividing N pixels into C classes, and the cluster center of each class is expressed as *Z* = {*z*_1_,*z*_2_,…,*z*_*N*_}. The functional expression of FCM is

(1) $$J_{F\,C\,M}=\sum_{i=1}^{C}\sum_{j=1}^{N}u_{i\,j}^{m}\,d^{2}\,\left(x_{j},z_{i}\right)$$

(2) $$d^{2}\,\left(x_{j},z_{i}\right)={||x_{j}-z_{i}||}^{2}$$

The size of *m* determines the clustering ambiguity. If the value of *m* is too large, the fuzziness of clustering will increase accordingly, which is not conducive to the defuzzification of the model. In contrast, the smaller the value of *m* is, the smaller the clustering ambiguity, and the segmentation result is close to hard segmentation. In practical applications, the value of *m* is usually taken as 2. Equations (3) and (4) give the iterative formula of cluster center *z*_*i*_ and membership degree *u*_*ij*_.

(3) $$z_{i}=\frac{\sum_{j=1}^{N}u_{i\,j}^{m}\,x_{j}}{\sum_{j=1}^{N}u_{i\,j}^{m}}$$

(4) $$u_{i\,j}=\frac{1}{\sum_{r=1}^{C}\left[{\left(\frac{d\,\left(x_{j},z_{i}\right)}{d\,\left(x_{j},z_{r}\right)}\right)}^{\frac{2}{m-1}}\right]}$$

**FIGURE 3:**
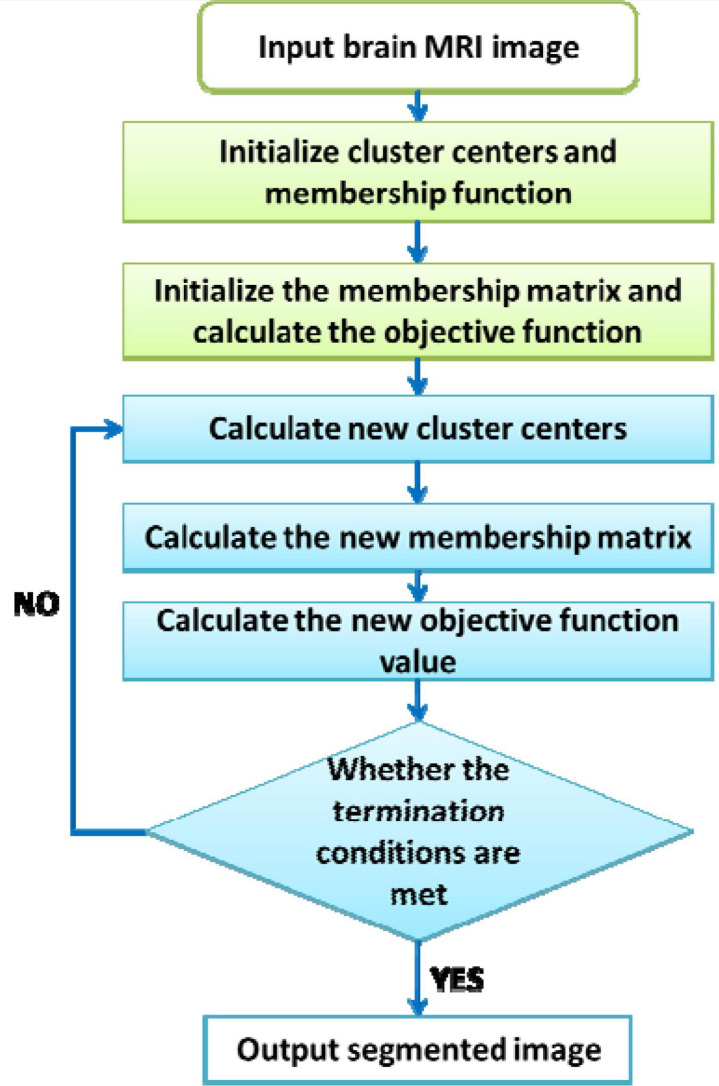
B-MRI-IS process based on FCM.

The steps of the FCM-based image segmentation algorithm are as follows: (1) Input the brain MRI image; (2) set the fuzzy factor *m* = 2, the maximum number of iterations ε (ε > 0), the number of clusters *C*, and initialize the cluster center and membership matrix randomly; (3) according to Eqs 3 and 4, update the clustering center *z* and membership degree *u*; (4) make the objective function converge until the cluster center no longer changes; and (5) output the segmented image. The flow of B-MRI-IS based on FCM is shown in Figure 3.

### Multiview Clustering Algorithm

Traditional fuzzy clustering cannot achieve ideal results when processing multiview data. Based on this requirement, many multiview-related algorithms have been proposed. The existing multiview-related clustering algorithms include coclustering (Gu and Zhou, 2009), collaborative clustering algorithms (Pedrycz, 2002), and multitask clustering algorithms (Chen et al., 2013). There are many multiview clustering algorithms, the most classic of which is CoFKM. Next, we will briefly introduce the principle and implementation steps of the algorithm. Table 4 gives the definition of each symbol of the algorithm.

**TABLE 4:** Description of related symbols in the multiview clustering algorithm.

Symbols	Description	Symbols	Description
*X* = {*view*_1_,*view*_2_,…,*view*_*V*_}	Multiview dataset	*view*_*v*_ = [*cpsbreak*]{*x*_1,*v*_,*x*_2,*v*_,…,*x*_*N*,*v*_}	*v*th view data
*N*	Total number of samples	*V*	Number of views
*C*(2≤*C*≤*N*)	Number of clusters	η	Parameters that regulate the importance of each view
*Z*_*v*_ = [*z*_1,*v*_,*z*_2,*v*_,…,*z*_*c*,*v*_]	Class center of the *v*th view	*U*_*v*_ = [*u*_*ij*,*v*_]	Membership of the *v*th view

Equation (5) gives the function expression of CoFKM:

(5) $$J_{C\,\text{o}\,F\,K\,M}=\sum_{v=1}^{V}\sum_{i=1}^{C}\sum_{j=1}^{N}{\tilde{u}}_{i\,j,v,\eta }\,{||x_{j,v}-z_{i,v}||}^{2}$$

(6) $${\tilde{u}}_{i\,j,v,\eta }=\left(1-\eta \right)\,u_{i\,j,v}^{m}+\frac{\eta }{V-1}\,\sum_{v^{\prime }=1,v^{\prime }\neq v}^{V}u_{i\,j,v^{\prime }}^{m}$$

(7) $$\left\{ \begin{array}{c} u_{i\,j,v}\in \left[0,1\right],1\leq i\leq C,1\leq j\leq N\\ \sum_{i=1}^{C}u_{i\,j,v}=1,1\leq v\leq V \end{array}\right.$$

where ${\tilde{u}}_{i\,j,v,\eta }$ represents the weighted fusion of the membership degree $u_{i\,j,v}^{m}$ under the current view and the membership degree $u_{i\,j,v^{\prime }}^{m}$ of the other views. According to Eq. 5, through the Lagrangian extremum solution method, the calculation formulas of the clustering center and membership degree are obtained. Equations (8) and (9) give the calculation formulas of the cluster center and membership matrix of the *v*th view.

(8) $$z_{i,v}=\frac{\sum_{j=1}^{N}{\tilde{u}}_{i\,j,v,\eta }\,x_{i,v}}{\sum_{j=1}^{N}{\tilde{u}}_{i\,j,v,\eta }},i=1,2,\ldots ,C$$

(9) $$u_{i\,j,v}=\frac{1}{\sum_{h=1}^{C}\left[\frac{\left(1-\eta \right)\,d_{i\,j,v}^{2}+\frac{\eta }{V-1}\,\sum_{v^{\prime }=1,v^{\prime }\neq v}^{V}d_{i\,j,v^{\prime }}^{2}}{\left(1-\eta \right)\,d_{h\,j,v}^{2}+\frac{\eta }{V-1}\,\sum_{v^{\prime }=1,v^{\prime }\neq v}^{V}d_{h\,j,v^{\prime }}^{2}}\right]}$$

Through Eqs 8 and 9, the division matrix for any view is calculated. The final division result is obtained using the ensemble method of the geometric mean. The expression of the integrated method is

(10) $$\overset{⌢}{U}=\sqrt[{V}]{\prod_{v=1}^{V}U_{v}}$$

### Algorithm Evaluation Index

The goal of segmentation is to divide pixels with certain characteristic properties (such as grayscale and spatial position) in an image into a region. All the pixels in a region have similar properties, and the characteristic properties of pixels in different regions are quite different. The evaluation index of the effectiveness of image segmentation is used to describe the pros and cons of the segmentation results. The commonly used evaluation indicators include segmentation accuracy *JS*, Dice coefficient (*DSC*), similarity (*KI*), segmentation coefficient (*V*_*pc*_), segmentation entropy (*V*_*pe*_), and pixel error rate (*ME*). The specific descriptions of commonly used evaluation indicators are shown in Table 5.

**TABLE 5:** Evaluation index descriptions.

Index	Description
$J\,S\,\left(S_{1},S_{2}\right)=\frac{\left|S_{1}\cap S_{2}\right|}{S_{1}\cup S_{2}}\,\left[c\,p\,s\,b\,r\,e\,a\,k\right]\,D\,S\,C\,\left(S_{1},S_{2}\right)=\frac{2\,\left|S_{1}\cap S_{2}\right|}{\left|S_{1}\right|+\left|S_{2}\right|}$	*S*_1_ represents the segmentation area and *S*_2_ represents the ground truth. The larger the *JS* value, the better the segmentation accuracy of the algorithm and the better the effect.
$\begin{array}{c} K\,I=\frac{T\,P}{\left(2\,T\,P+F\,P+F\,N\right)/2}\\ =\frac{2\,T\,P}{2\,T\,P+F\,P+F\,N} \end{array}$	True positive (*TP*), false positive (*FP*), false negative (*FN*); *KI* is a value greater than 0 and less than 1. *KI* = 1 means that the algorithm segmentation result is completely consistent with the standard segmentation. The closer the *KI* is to 1, the more accurate the segmentation result and the better the algorithm performance.
$V_{p\,c}=\frac{\sum_{j=1}^{n}\sum_{i=1}^{c}u_{i\,j}^{2}}{n}\,\left[c\,p\,s\,b\,r\,e\,a\,k\right]\,V_{p\,e}=\frac{\sum_{j=1}^{n}\sum_{i=1}^{c}\log u_{i\,j}}{n}$	These two segmentation indexes are related to the degree of membership *u*_*ij*_. Generally, a segmentation model with less ambiguity is considered to have better performance. The closer *V*_*pc*_ is to 1, and the closer *V*_*pe*_ is to 0, the higher the segmentation accuracy of the algorithm.
*ME*(*A*,*B*) = $\frac{A\,r\,e\,a\,\left(S_{A}\cup S_{B}\right)-A\,r\,e\,a\,\left(S_{A}\cap S_{B}\right)}{A\,\text{rea}\,\left(S_{B}\right)}$	The segmented image is compared with the “gold standard” image, and the ME. *S*_*A*_ of the obtained model represents the target area extracted by the segmentation algorithm, and *S*_*B*_ represents the “gold standard” target area. The value range of *ME* is 0 to infinity. The closer *ME* is to 0, the smaller the mis-segmentation area and the higher the segmentation accuracy.

### Experimental Dataset

Common brain image databases are mainly as follows: Brain Perfusion Database, Allen Brain Atlas, BRATS database, simulated brain MR image data from the Brain Web website, and the Internet Brain Segmentation Repository (IBSR). The description of each database is shown in Table 6.

**TABLE 6:** Brain image database.

Database	Image size	Classification details
Brain Perfusion Database (Cleuziou et al., 2009)	194*237	Number of clusters: 4, which are WM, GM, CSF, and background
Allen Brain Atlas (Okita et al., 2015)	256*128	
BRATS (Rosati et al., 2018)	155*240*240	
Brain Web (Weijer and Gevers, 2006)	181*217*181	
IBSR (Liu et al., 2020)	256*256	

“*” Represents multiplication.

## Brain MRI Image Segmentation Based on Multiview Fuzzy Clustering

### Brain MRI Image Segmentation Process Based on Multiview Clustering

**FIGURE 4:**
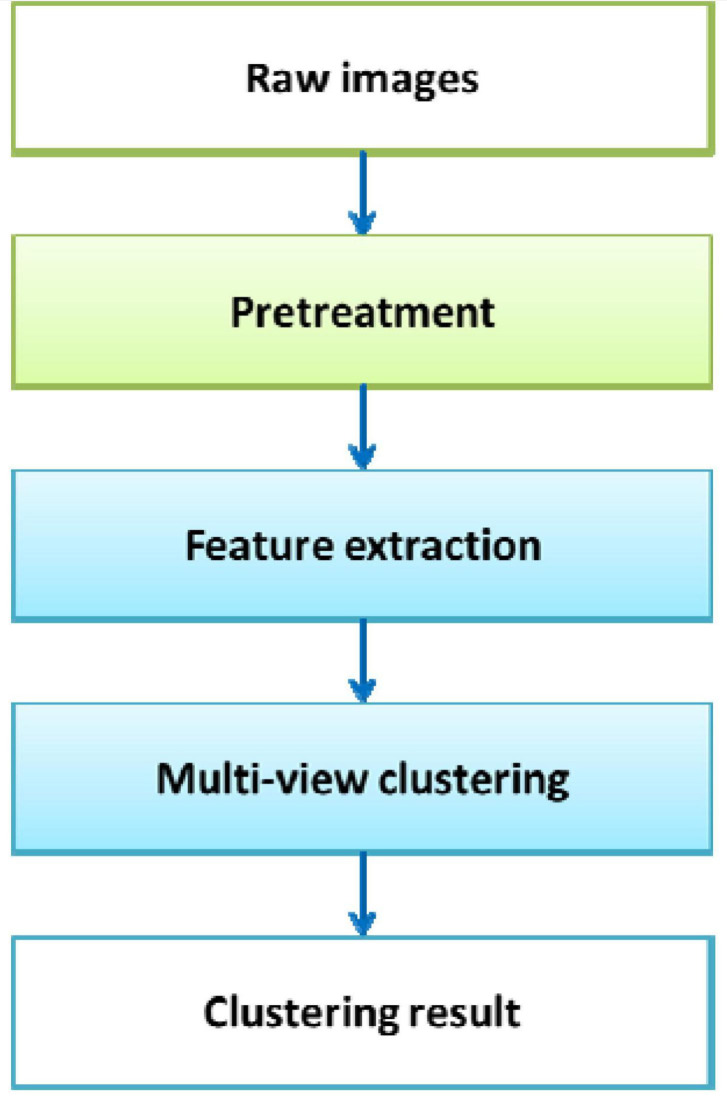
B-MRI-IS process based on multiview clustering.

MVD carries more information than single-view data. This MVD feature is beneficial to the improvement of cluster analysis. For clustering analysis of MVD, the traditional single-view clustering algorithm is to first separate each view data from the MVD. Second, the single-view clustering algorithm is used to process the data of each view, and the clustering results of the data of each view are obtained. Finally, a suitable ensemble strategy is used to fuse the clustering results of all views to obtain the final division result. The single-view clustering algorithm for processing MVD has the following shortcomings. When the clustering results of a certain view have obvious deviations or the clustering results between different views are very different, if each view is artificially separated for analysis in this way, the final division result obtained by integrated learning is likely to be poor or the performance of the algorithm is unstable. To make full use of the information carried by MVD and improve the accuracy of clustering, this research introduces multiview technology into the single-view clustering method. The multiview clustering algorithm enables the collaborative learning of various views in the clustering process, makes full use of the data information of each view, and improves the clustering performance and stability of the algorithm. The B-MRI-IS process based on multiview clustering is shown in Figure 4.

As shown in Figure 4, first, the original brain image is preprocessed, such as denoising. Second, the HOG feature, entropy feature, gradient feature, and contrast feature of the original image are extracted. The feature data serve as view data. Third, all view data are input into the multiview clustering model to obtain the final clustering result. Due to the different amounts of information contained in different features, the contribution of the formed view data to the clustering result is also different. Therefore, how to effectively adjust the weight of each view is very important during the use of the multiview clustering algorithm.

### IMV-FCM

The CoFKM introduced in the section “Multiview Clustering Algorithm” assigns the same weight to each view, which shows that the CoFKM believes that the contribution of each view data to the clustering is the same. This is obviously inconsistent with the actual situation. For MVD in real production and living environments, the contribution of each view data to the clustering is necessarily different. If the angle of view data with a high degree of contribution is given a high weight and the angle of view data with a low degree of contribution is given a low weight, the overall clustering performance can be improved. Therefore, a multiview fuzzy clustering method with adaptive viewing angle adjustment capability is used in this study, and its objective function is

(11) $$\begin{array}{c} J=\sum_{v=1}^{V}\sum_{i=1}^{C}\sum_{j=1}^{N}\sum_{t=1}^{V}w_{v,t}\,u_{i\,j,t}^{m}\,d_{i\,j,v}^{2}+\gamma \,\sum_{v=1}^{V}\sum_{t=1}^{V}w_{v,t}\,\log\left(w_{v,t}\right)\\ \sum_{i=1}^{C}u_{i\,j,v}=1,u_{i\,j,v}\in \left[0,1\right]\\ 1\leq i\leq C,1\leq j\leq N,1\leq v\leq V\\ \sum_{t=1}^{V}w_{v,t}=1,w_{v,t}\in \left[0,1\right],1\leq v\leq V \end{array}$$

where $d_{i\,j,v}^{2}={||x_{j,v}-z_{i,v}||}^{2}$ and *U*_*v*_ = [*u*_*ij*,*v*_] is the division matrix corresponding to the *i*th view. ${\tilde{u}}_{i\,j,v}=\sum_{t=1}^{V}w_{v,t}\,u_{i\,j,t}^{m}$ is the division fusion item, which realizes the view fusion of different views in the *v*th view clustering task. At this time, the importance of each view is reflected by the weight *w*_*v*,*t*_. *w*_*v*,*t*_ represents the importance of the *t*th view in the *v*th view cluster. *W* is the weight matrix of all views. The Lagrange multiplier method is used to solve the extreme value of Eq. 11, and the expression of each variable is obtained as follows:

(12) $$z_{i,v}=\frac{\sum_{j=1}^{N}\sum_{t=1}^{V}w_{v,t}\,u_{i\,j,t}^{m}\,x_{j,v}}{\sum_{j=1}^{N}\sum_{t=1}^{V}w_{v,t}\,u_{i\,j,t}^{m}}$$

(13) $$u_{i\,j,t}=\frac{1}{\sum_{h=1}^{C}{\left[\frac{\sum_{v=1}^{V}w_{v,t}\,d_{i\,j,v}^{2}}{\sum_{v=1}^{V}w_{v,t}\,d_{h\,j,v}^{2}}\right]}^{1/\left(m-1\right)}}$$

(14) $$w_{v,t}=\frac{\exp\left[\frac{-\sum_{i=1}^{C}\sum_{j=1}^{N}u_{i\,j,t}^{m}\,d_{i\,j,v}^{2}}{\gamma }\right]}{\sum_{g=1}^{V}\exp\left[\frac{-\sum_{i=1}^{C}\sum_{j=1}^{N}u_{i\,j,g}^{m}\,d_{i\,j,v}^{2}}{\gamma }\right]}$$

The following ensemble method is used to obtain the final partition matrix:

(15) $$\bar{U}=\max\left(\sum_{t=1}^{V}\left(\frac{\sum_{v=1}^{V}w_{v,t}}{\sum_{t=1}^{V}\sum_{v=1}^{V}w_{v,t}}\,U_{t}\right)\right)$$

**FIGURE 5:**
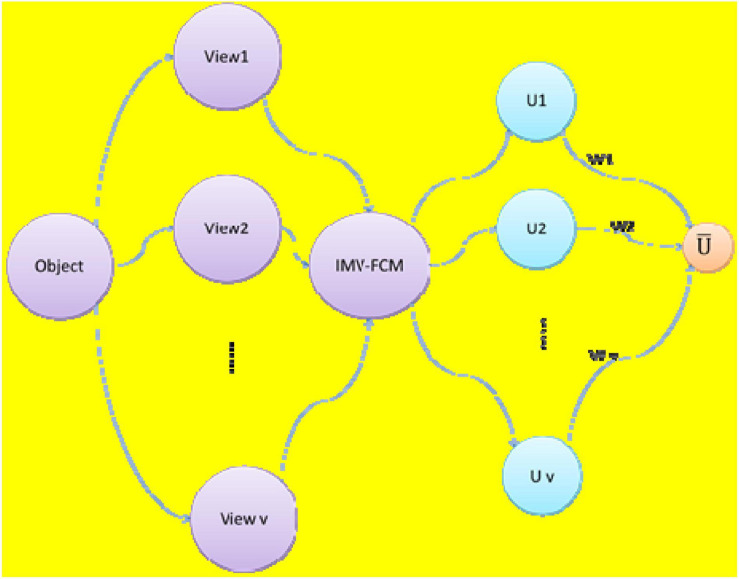
Schematic diagram of IMV-FCM algorithm.

Figure 5 shows the schematic diagram of the IMV-FCM algorithm. As shown in Figure 5, after the multiview data with *v* views are executed by the IMV-FCM algorithm, *v* membership matrices are obtained. Each membership matrix is assigned a corresponding weight *w*, and the final global membership matrix is obtained according to the fusion strategy of Eq. 15.

The specific steps of the IMV-FCM algorithm are in Algorithm 1.

**Algorithm 1:** IMV-FCM algorithm.

Input	Multiview sample set *X*, number of views *V*, number of clustering *C*, iteration threshold ε, fuzzy index *m*, iteration number *l*, parameter γ.
Output	The final division matrix $\bar{U}$, the clustering center of each view *z*_*i*,*v*_, the view fusion weight matrix *W* = {*w*_*v*,*t*_}.
Step 1	Randomly generate fuzzy membership matrix *u*_*ij*,*t*_(1≤*t*≤*V*) for each view and view fusion weight matrix *W* = {*w*_*v*,*t*_} for each view;
Step 2	According to Eq. 12, the cluster center *z*_*i*,*v*_ of each view is updated.
Step 3	According to Eq. 13, the membership degree *u*_*ij*,*t*_ of each view is updated.
Step 4	According to Eq. 14, the view fusion weight matrix *W* = {*w*_*v*,*t*_} is updated.
Step 5	If ||*J*^*l* + 1^−^*Jl*^|| < ε, the algorithm stops iterating; otherwise, it returns to Step 2.
Step 6	After the algorithm converges, the fuzzy membership of each view is output.
Step 7	According to the fuzzy membership degree of each view obtained in Step 6, Eq. 15 is used to obtain the final division matrix.

## Simulation Experiment Analysis

### Experimental Background

The dataset used in this study was the data downloaded from BrainWeb. The 2D images of 89 cross sections (89 CS), 92 cross sections (95 CS), and 95 cross sections (95 CS) in the T1-weighted brain MRI image were selected for segmentation. The comparison algorithms used were FCM, CoFKM (Cai et al., 2019), two-layer automatic weighted clustering algorithm (TW-k-means) (Singh et al., 2020), multitask-based K-means (CombKM) (Chen et al., 2013), and collaborative clustering based on sample and feature space (coclustering) (Gu and Zhou, 2009). In the experiment, the iteration stop threshold ε of each algorithm is set to 0.001, and the maximum number of iterations *l* was set to 100. The parameter settings of each algorithm are shown in Table 7. The parameter selection methods of each comparison algorithm include the grid search method and clustering index. This article mainly uses the grid search method to confirm the optimal parameters. The reason for choosing the grid search method for parameter selection is that the performance of the model trained based on the parameters selected by this method is the best. The evaluation index adopts JS and DSC. All experiments were based on the MATLAB 2018a programming environment, which was simulated on a Lenovo PC configured with a Windows 10 operating system, 2.60 GHz CPU, and 8G memory Intel(R) Core(TM)i7 processor.

**TABLE 7:** Parameter setting of various algorithms based on grid search.

Algorithm	Parameter setting range
FCM	Fuzzy factor *m* = {1.05,1.1,1.2,1.3,1.4,1.5,1.6,1.7,1.8,1.9,2}
CoFKM	Fuzzy factor *m* = {1.05,1.1,1.2,1.3,1.4,1.5,1.6,1.7,1.8,1.9,2}, collaborative learning parameters η = [0,(*K*−1)/*K*], *K* is the number of views
TW-*k*-means	Regularization parameters λ = {1,2,…,30} and η = {10,20,…,100}
CombKM	None
Coclustering	Regularization parameter λ = {1,10,100,300,500,800,1000}, regularization parameter μ = {1,10,100,300,500,800,1000}, feature category number *m* = ⌊d/2⌋, *d* is the characteristic number, ⌊⌋ means rounding down.
IMV-FCM	Fuzzy factor *m* = {1.05,1.1,1.2,1.3,1.4,1.5,1.6,1.7,1.8,1.9,2} Regularization parameters γ = {2^−12^,2^−11^,…,2^12^}

### Experimental Results and Analysis

To verify the superiority of the IMV-FCM algorithm, the experiment section gives a comparison of the effect of each algorithm on image segmentation. In analyzing the antinoise performance of the algorithm, this study adds different proportions of Gaussian noise to the image. The segmentation results of each algorithm are shown in Tables 8, 9. The symbol A represents WM, B represents GM, and C represents CSF.

**TABLE 8:** JS indicators of each algorithm (100%).

Noise	Organization	FCM	CoFKM	TW-k-means	CombKM	Coclustering	IMV-FCM
0%	A	85.73%	88.22%	87.76%	87.85%	89.96%	90.45%
	B	74.52%	77.85%	76.90%	77.11%	79.93%	79.76%
	C	65.98%	68.31%	67.58%	68.32%	69.94%	70.74%
	Mean	75.41%	78.13%	77.41%	77.76%	79.94%	80.32%
3%	A	84.98%	87.52%	88.65%	87.03%	87.67%	89.20%
	B	73.87%	76.48%	76.77%	75.96%	76.35%	78.16%
	C	64.76%	66.54%	67.12%	65.87%	66.11%	67.91%
	Mean	74.54%	76.85%	77.51%	76.29%	76.71%	78.42%
5%	A	84.17%	86.11%	86.59%	85.90%	85.97%	87.79%
	B	73.25%	75.43%	75.86%	74.97%	74.98%	76.58%
	C	64.88%	66.35%	66.36%	65.89%	66.99%	67.02%
	Mean	74.10%	75.96%	76.27%	75.59%	75.98%	77.13%
7%	A	82.21%	84.46%	82.32%	82.98%	82.12%	85.80%
	B	72.62%	74.02%	73.52%	73.87%	73.48%	75.35%
	C	64.14%	66.62%	65.07%	65.85%	65.17%	67.56%
	Mean	72.99%	75.03%	73.64%	74.23%	73.59%	76.24%
9%	A	76.68%	82.23%	80.61%	81.02%	81.43%	83.56%
	B	70.25%	73.18%	71.17%	71.65%	71.98%	72.20%
	C	62.27%	65.83%	64.59%	64.04%	64.47%	71.80%
	Mean	69.73%	73.75%	72.12%	72.24%	72.63%	75.85%

**TABLE 9:** DSC indicators of each algorithm (100%).

Noise	Organization	FCM	CoFKM	TW-k-means	CombKM	Coclustering	IMV-FCM
0%	A	93.24%	94.75%	94.28%	93.39%	94.02%	95.27%
	B	86.51%	87.79%	87.61%	86.87%	87.99%	88.51%
	C	80.45%	82.44%	82.83%	82.10%	83.04%	85.06%
	Mean	86.73%	88.33%	88.24%	87.45%	88.35%	89.61%
3%	A	92.28%	93.68%	93.77%	93.91%	94.23%	95.08%
	B	85.30%	86.84%	87.04%	86.89%	86.57%	87.62%
	C	79.47%	82.02%	83.22%	82.16%	83.01%	84.41%
	Mean	85.68%	87.51%	88.01%	87.65%	87.94%	89.04%
5%	A	91.36%	91.03%	91.14%	91.09%	91.74%	93.86%
	B	84.89%	84.47%	84.66%	84.82%	84.96%	86.68%
	C	78.55%	80.10%	80.23%	80.34%	80.08%	83.71%
	Mean	84.93%	85.20%	85.34%	85.41%	85.59%	88.08%
7%	A	89.62%	90.02%	90.91%	90.54%	90.74%	92.95%
	B	84.01%	83.36%	83.82%	83.57%	84.10%	85.45%
	C	77.86%	79.03%	79.65%	79.66%	80.12%	82.79%
	Mean	83.83%	84.14%	84.79%	84.59%	84.99%	87.06%
9%	A	85.76%	88.44%	88.62%	88.79%	89.76%	91.87%
	B	83.79%	81.45%	81.89%	81.63%	82.30%	83.67%
	C	77.23%	77.72%	77.79%	77.87%	78.31%	81.32%
	Mean	82.26%	82.54%	82.77%	82.76%	83.46%	85.62%

From the experimental data in Tables 8, 9, it can be determined that when processing original brain images, the segmentation performance of the multiview algorithm used in this study is significantly better than FCM. This shows that the introduction of the multiview mechanism can mine information from multiple views, thereby improving the clustering accuracy. Compared with other multiview clustering algorithms, the segmentation effect of the IMV-FCM algorithm in this study is better. This shows that the IMV-FCM algorithm with partition adaptive fusion capability requires the Co-FKM algorithm and CombKM algorithm to manually set the degree of partition fusion. The IMV-FCM algorithm can obtain better view weights during multiview learning and finally obtain better multiview clustering effects.

**FIGURE 6:**
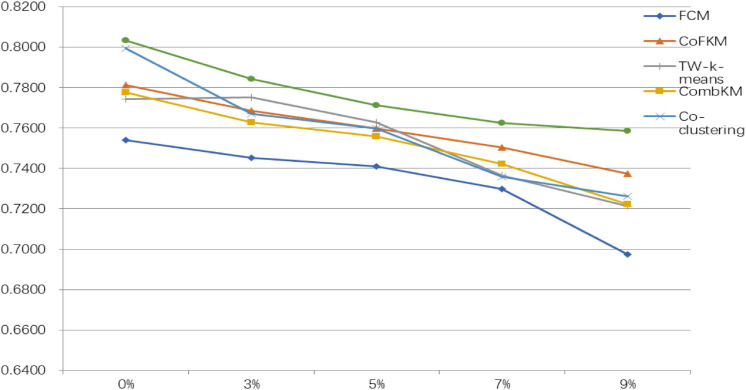
JS indicator drop rate of each algorithm.

**FIGURE 7:**
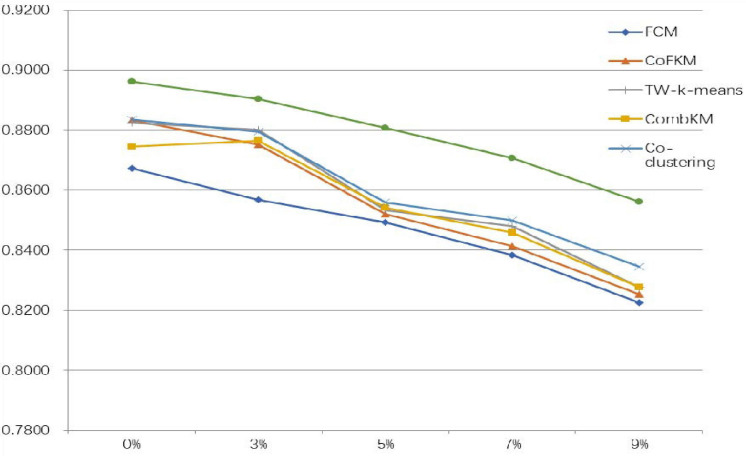
DSC index drop rate of each algorithm.

As the image noise increases, the clustering performance of all clustering algorithms begins to decline. However, even if the performance of each algorithm is declining, the performance of the IMV-FCM algorithm is still better than that of the other algorithms. This is manifested in two aspects. First, the clustering performance of IMV-FCM is better than that of the comparison algorithm. The second is that with the increase in image noise, the clustering performance of the IMV-FCM algorithm decreases the most slowly, as shown in Figures 6, 7. This fully shows that IMV-FCM is more robust to noise.

## Conclusion

To further improve the performance of B-MRI-IS, the IMV-FCM algorithm is applied in this research. The IMV-FCM algorithm is a multiview fuzzy clustering algorithm with adaptive view weight. Under the adaptive learning of the view fusion weight matrix, the coordination between various views is more flexible. Additionally, each view can be self-adapted to learn and then achieve a better clustering effect. Since the original brain MRI image is not MVD, this study extracts multiple feature data of the original image and uses one feature data as one view data to construct MVD. The experimental results prove that the segmentation method used in this paper optimizes the segmentation effect. With the increase in noise, the clustering effect of IMV-FCM decreases more slowly than the comparison method, which shows that the method used has stronger noise immunity. Although the effectiveness of IMV-FCM was verified, it still faces certain limitations. For example, the proposed method uses the classic FCM algorithm as a framework and uses Euclidean distance, which causes it to suffer from the dimensionality disaster problem when facing high-dimensional multiview clustering tasks. How to solve this problem will be the focus of future research. In addition, similar to most unsupervised learning algorithms, the selection of optimal parameters is an important issue. Different practical applications require different ranges of parameter values. Since the optimal parameters are usually determined by the application, for an exact application, the appropriate parameter range of the algorithm used can be determined through prior knowledge or available valid labeled datasets. The ensemble learning strategy can avoid the selection of optimal parameters to a certain extent, and it is planned to be further studied in follow-up work.

## Data Availability Statement

Publicly available datasets were analyzed in this study. This data can be found here: https://brainweb.bic.mni.mcgill.ca.

## Author Contributions

LH and YG conceived and developed the theoretical framework of the manuscript. YG and XG designed and performed the surgical procedure of the case presentation. LH, JX, and TN performed the data evaluation, analysis, and interpretation, designed the figures, designed and wrote the manuscript in consultation with YG and XG, who took the lead in writing the manuscript. All authors participated in the editing process.

## Conflict of Interest

The authors declare that the research was conducted in the absence of any commercial or financial relationships that could be construed as a potential conflict of interest.
